# Mineralogical Analysis of Historical Mortars by FTIR

**DOI:** 10.3390/ma12010055

**Published:** 2018-12-24

**Authors:** M. M. Jordán, J. Jordá, F. Pardo, M. A. Montero

**Affiliations:** 1 Department of Agrochemistry and Environment, Miguel Hernández University of Elche, 03202 Elche (Alicante) Spain; adrianaidiomas@umh.es; 2 Multidisciplinary Institute for Environmental Research “Ramon Margalef”, University of Alicante, 03080 Alicante, Spain; juana.jorda@ual.es; 3 Department of Education, University CEU Cardenal Herrera, 12006 Castellón, Spain; francisco.pardo@uch.ceu.es

**Keywords:** mineralogical analysis, historical mortars, FTIR

## Abstract

A method for quantitative mineralogical analysis by ATR-FTIR [1] has been used first time for analysis of historical mortars. Mixtures of different minerals and gypsum were used in order to measure the minimum band intensity that must be considered for calculations and the detection limit. In this way, the molar absorptivity coefficient in the Lambert–Beer law and the components of a mixture in mol percentage can be calculated. The GAMS equation modeling environment and the NLP solver CONOPT (©ARKI Consulting and Development) were used to correlate the experimental data in the samples considered. The characterization of the vernacular mortars by FTIR analysis identifies the predominant minerals of the samples, and in conjunction with XRF and XRD, shows the exact composition of historical mortars, which will optimize the restoration and conservation of monuments, preserving our heritage.

## 1. Introduction

The need for new, cheap, and fast analysis techniques has led to research in new technologies, particularly those based on spectroscopic methods. Mineralogical characterization of complex samples, such as clays, soils, or pottery, is a main topic in order to determine their physico-chemical characteristics and functionality [1]. ATR (attenuated total reflectance) is a spectroscopic method frequently used with IR spectrophotometers (absorption recording method). This technique has been widely applied in mineralogical studies [2] including ancient pottery [3] and ceramics [1]. Nevertheless, in media as complex as these, problems remain such as the extensive use of blanks in order to properly quantify samples. In aqueous media, calculating the molar absorptivity of the Lambert–Beer law is easy, and it can be used for component concentration calculations since an aqueous, constant concentration matrix exists. In solid media, such as soils, clays, mortars, or ceramics, this may not occur and the problem is more complicated, having to take into account that the main components are silicates that provide signals within the same wavenumber range [1].

In this paper, an approach for calculating the relative intensities of each mineral species in a mixture when they are measured by ATR-FTIR is proposed, comparing the intensities of the major bands of different minerals [1] with the main band of calcite, since the overlap between the main calcite band (1380 cm^−1^) and the main bands of other minerals is minimum [1].

## 2. Materials and Methods

The study sampling area is the Penyagolosa massif, in the province of Castellón (Spain), from where we took five representative samples of mortars from Historical Centres and surrounding buildings (farmhouses and mills) following the recommendations in [4]. These samples were analysed mainly by FTIR following the procedure described in [1]. The objective of this study was to characterize vernacular mortars by FTIR analysis using X-ray fuorescence (XRF) and X-ray diffraction (XRD) as standard techniques in order to characterize them. Orientated clay aggregates (normal, heated to 550 °C for 2 h and treated with ethylene glycol for 2 h) were also prepared. X-ray spectra were recorded using a Siemens D-500 diffractometer with Bragg–Bretano geometry (Siemens, Wien, Austria). A normal diagram from 4–70° (2θ) was made using the Siemens software package DIFFRACT-AT version 3.1 (Siemens, Wien, Austria). Chemical analysis of raw materials was carried out by XRF using the conventional techniques.

For the FTIR analysis, a fine powder of the different mineral samples was placed with no further treatment on the diamond window of the ATR-FTIR instrument spectrometer (BRUKER IFS 66/S) (BRUKER, Karlsruhe, Germany). Different blanks from the mineralogical collection of our lab were used, including silicates, sulfates, carbonates, nitrates, and phosphates. The samples analyzed were pure minerals (crystalline samples and lab substances) or mineral mixtures used as standards in order to prove the quality of the analysis. Due to the range of λ, the majority of oxides are not detected by this method. Calcite and gypsum (Panreac™, RA) were used as references. The raw spectra were processed for the baseline and normalized as described in [2] using Excel software (Microsoft^®^). Obtaining ε values for each mineral is complicated, since in most cases the main bands overlap. To approach signal differences, and therefore mineral concentrations in samples, equimolar mixtures of different minerals and calcite (Panreac, purity 98%) were analyzed. The main calcite band (1400 cm^−1^) appears quite distant from silicate, phosphate, or sulfate bands (900–1000 cm^−1^). Silicate, phosphate, or sulfate spectra overlap each other and therefore cannot be used as a reference. An organic or inorganic substance with a single IR spectrum and good signal absorption bands, which do not overlap with those of other minerals, is needed. The GAMS equation modeling environment and the NLP solver CONOPT (©ARKI Consulting and Development) were used to correlate the experimental data in the samples considered. Calculation of the mineral content in each sample was conducted by solving:

$$I=\sum_{j=1}^{a}{\mathrm{ii}}_{j}$$

where I is the signal intensity at a wavenumber (λ); i, the signal of each component at that λ; and a, the number of components in the sample. According to the Lambert–Beer law, the signal intensity of each component is proportional to its concentration.

Five representative samples of mortars from Historical Centres, farm houses, and mills were also analyzed. These samples were characterized previously by X-ray Fluorescence (chemical analysis) and by XRD. Semi-quantitative mineralogical analysis was carried out following the methodology in [5].

## 3. Results and Discussion

**Figure 1:**
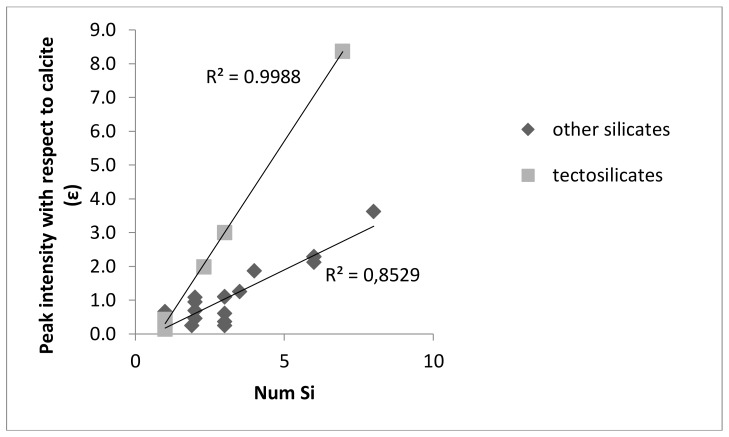
Main peak intensity in the IR range of 600–4000 cm^−1^ with respect to the calcite main peak for several silicates as a function of the number of Si atoms in the formula.

**Figure 2:**
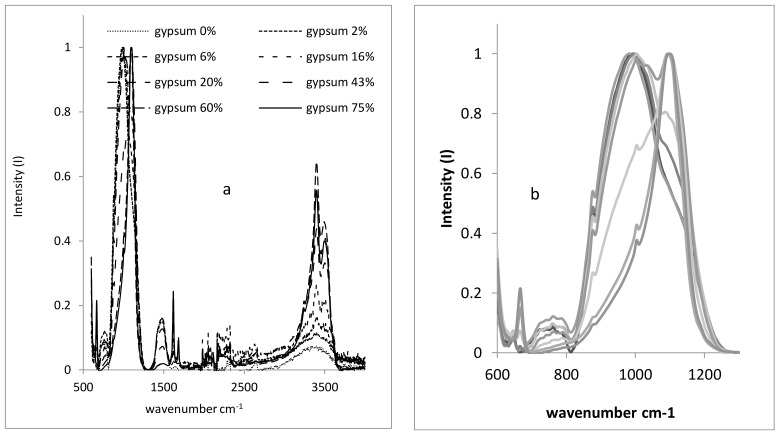
FTIR spectra of several clay–gypsum mixtures (% weight). Total spectrum (**a**), overlapped bands silicate–sulphate (**b**).

The main band intensities of several silicates with respect to calcite are a function of the number of Si atoms in the formula (Figure 1 and Figure 2).

The samples analyzed (Table 1 and Table 2) clearly show two types of mortar (lime or gypsum), as well as the mineralogy of the mixture and the inorganic binder that originally constituted the mortar. Sample 1 has a high percentage of halloysite, and lower amounts of calcite and apatite. The XRF of this sample coincides with the FTIR analysis, showing high amounts of CaO and SiO_2_. The chemical analysis shows the existence of P_2_O_5_ and Cl^−^ from these same minerals, as well as SO_3_ y Na_2_CO_3_ related to the hanksite. In regards to sample 2, it is worth noting that it is a gypsum mortar. Basanite was formed during the transformation of anhydrite into gypsum, possibly due to poor baking and the climatic factors that have taken place since the mortar was produced. Furthermore, this second sample also contained feldspars, which we know are albite and orthoclase thanks to the XRD.

The third sample is similar to the second one, although the percentage of SO_3_ and orthoclase is slightly superior. Regarding the fourth sample, it is a lime mortar with prevailing amounts of calcite, followed by halloysite and celestine. The XRF shows the prevalence of CaO and SiO_2_ along with NaO_2_ due to the presence of hanksite. The fifth sample is a lime mortar with clear traces of SiO_2_ in the sands used for its production. The XRD of this sample shows an important amount of quartz and calcite, as well as orthoclase and illite. Both techniques (XRD and FTIR) were similar in the detection of the same minerals. However, the detection limits and the sensitivity for each mineral are quite different. XRD patterns are dominated by quartz, unlike the FTIR spectra, where this mineral was more difficult to detect. In FTIR, some compounds with similar spectra can be difficult to solve. On these occasions, XRD remains a key support technique because of its ability to discriminate between different crystal structures.

## 4. Conclusions

The characterization of the vernacular mortars with an FTIR analysis identifies the predominant minerals of the samples, and in conjunction with XRF and XRD, shows the exact composition of historical mortars, which will optimize the restoration and conservation of monuments, preserving our heritage. Differences in light absorption due to the molecular composition are not usually taken into account. One of the goals of this paper was to develop a method for calculating such differences that can be applied to other spectroscopic techniques that use solid samples such as mortars because it allows the use of spectra libraries and linear regression algorithms for quantification purposes. The main research highlights of this communication are: (a) A method for quantitative mineralogical analysis by ATR- FTIR has been developed; (b) This method uses relative absorbances relative the main absorbances of calcite; and (c) ATR-FTIR method was applied to mineral mixtures and historical mortars. However, direct measurements of (ε) for all minerals and research on matrix effects are needed to improve the analysis.

## Figures and Tables

**Table 1:** Chemical analysis by XRF (%) of five historical mortars.

%	AI_2_O_3_	SiO_2_	P_2_O_5_	SO_3_	K_2_O	CaO	MnO	Fe_2_O3	Na_2_O	Cl^−^
1	2.12	7.19	0.08	1.59	0.91	49.4	0.03	2.02	0.09	0.11
2	3.61	8.98	-	38	1.34	34.4	0.01	1.48	-	-
3	3.54	9.73	-	40	1.64	33.9	0.03	1.53	-	-
4	6.91	23.2	-	0.36	4.54	44.9	-	3.02	0.50	1.34
5	7.11	23	-	0.07	2.49	39.8	0.28	13.1	-	-

**Table 2:** Mineralogical composition (%) of five historical mortars (%) obtained by FTIR.

%	1	2	3	4	5
Quartz	4.90	0.69	0.69	-	2.03
Calcite	11.86	1.34	1.34	25.35	39.06
Halloysite	43.92	13.86	13.86	13.25	14.30
Apatite	11.03	-	-	-	8.83
Na_2_CO_3_	6.73	-	-	-	12.34
Feldspars	4.54	18.65	18.65	10.84	3.31
Hematite	4.59	3.92	3.92	7.44	5.69
Hanksite	6.97	-	-	8.45	-
Hornblende	3.34	-	-	-	-
Mn dioxide	-	3.36	3.36	-	-
Zeolite	-	10.21	10.21	-	-
Apophylite	-	2.91	2.91	-	3.03
Basanite	-	32.46	32.46	9.85	-
Magnesite	-	2.39	2.39	-	-
Jadeite	-	8.33	8.33	-	-
Celestine	-	-	-	13.52	-
Kaolinite	-	-	-	-	1.56
